# The Role of Circular RNAs in the Drug Resistance of Cancers

**DOI:** 10.3389/fonc.2021.790589

**Published:** 2022-01-05

**Authors:** Xin-Yuan Liu, Qi Zhang, Jing Guo, Peng Zhang, Hua Liu, Zi-Bin Tian, Cui-Ping Zhang, Xiao-Yu Li

**Affiliations:** Department of Gastroenterology, The Affiliated Hospital of Qingdao University, Qingdao, China

**Keywords:** circular RNAs, drug resistance, cancer, chemotherapy, therapeutic targets

## Abstract

Cancer is a major threat to human health and longevity. Chemotherapy is an effective approach to inhibit cancer cell proliferation, but a growing number of cancer patients are prone to develop resistance to various chemotherapeutics, including platinum, paclitaxel, adriamycin, and 5-fluorouracil, among others. Significant progress has been made in the research and development of chemotherapeutic drugs over the last few decades, including targeted therapy drugs and immune checkpoint inhibitors; however, drug resistance still severely limits the application and efficacy of these drugs in cancer treatment. Recently, emerging studies have emphasized the role of circular RNAs (circRNAs) in the proliferation, migration, invasion, and especially chemoresistance of cancer cells by regulating the expression of related miRNAs and targeted genes. In this review, we comprehensively summarized the potential roles and mechanisms of circRNAs in cancer drug resistance including the efflux of drugs, apoptosis, intervention with the TME (tumor microenvironment), autophagy, and dysfunction of DNA damage repair, among others. Furthermore, we highlighted the potential value of circRNAs as new therapeutic targets and prognostic biomarkers for cancer.

## Introduction

Cancer is a worldwide public health problem and a leading cause of premature death (1). The main reasons for the high mortality of patients and poor cancer prognosis are late diagnosis, tumor invasion and metastasis, and resistance to chemotherapeutic drugs. Resistance to chemotherapy, which involves intrinsic resistance and acquired resistance, has become a serious obstacle towards cancer therapy (2).

Although recent breakthroughs in treatment have contributed to the decline in cancer mortality, such as combined administration of drugs, checkpoint blockade immunotherapies, and targeted therapies for malignant tumors, tumor cells still exhibit a tendency towards drug resistance (1, 3). This becomes problematic as cancer cells become cross-resistant to several drugs with different anti-tumor mechanisms, causing invalid effects of various combination chemotherapies (4). Immune checkpoint inhibitors including programmed cell death 1 (PD1), PD1 ligand 1 (PD- L1), and cytotoxic T lymphocyte antigen 4 (CTLA4) have been successfully used in clinical applications. However, resistance to immune checkpoint blockade appeared simultaneously. For instance, it has been found that STK11/LKB1 mutations act as the main driver of immune escape and intrinsic resistance to PD-1 blockade in KRAS-mutant lung adenocarcinoma (LUAD) (5). Additionally, epidermal growth factor receptor (EGFR) tyrosine kinase inhibitors (TKIs) have been extensively studied as key targeted therapies, and within one or two years after treatment with TKIs, patients with activating alterations of the EGFR gene often acquire resistance to TKI therapy (2). Therefore, an insight into the specific molecular mechanisms that mediate drug resistance is crucial for understanding and overcoming drug tolerance in cancers.

**Figure 1:**
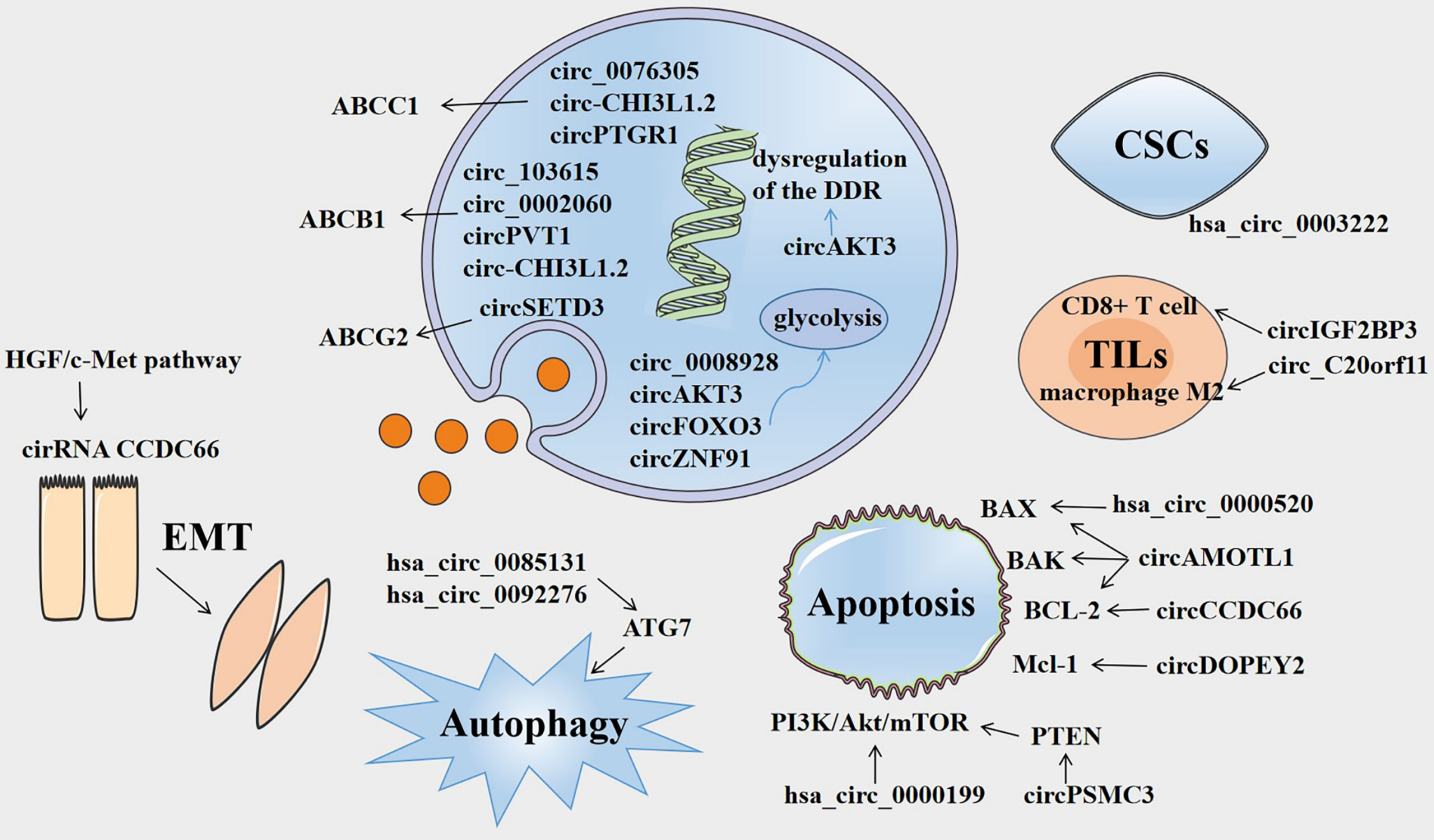
Overview of the involvement of circRNAs in cancer drug resistance. Several circRNAs are involved in drug resistance by influencing ABC efflux transporters, dysregulation of DNA damage response (DDR), glycolysis, epithelial-mesenchymal transition (EMT), autophagy, apoptosis, tumor-infiltrating lymphocytes (TILs), and cancer stem cells (CSCs).

Circular RNAs (circRNAs) were first identified in 1976 and belong to a growing list of types of non-coding RNAs, with a circular loop structure (6, 7). Most circRNAs, comprising a single exon or multiple exons, are expressed by known protein-coding genes (8). circRNAs can modulate gene splicing of pre-RNA and transcription, regulate RNA-binding proteins, and act as microRNA (miRNA) “sponges” to play a crucial role in transcriptional regulation (9, 10). Recent studies have revealed that numerous circRNAs are differentially expressed in various cancer patients and are correlated with the tumorigenesis and progression of cancers (11). For example, autophagy-associated circCDYL promotes the malignant progression of breast cancer cells and suppresses the clinical response to chemotherapy in patients with breast cancer (12). Conversely, circ-HuR suppresses the expression of CNBP-facilitated HuR and the progression of gastric cancer, serving as a tumor inhibitor (13). Moreover, accumulating evidence has shown that circRNAs are correlated with tumor chemoresistance and may play a crucial role in the occurrence and regulation of chemoresistance (14). To investigate the emerging role of circRNAs in the drug resistance of cancers, we systematically and comprehensively summarized the major mechanisms of cancer chemoresistance and the molecular mechanisms by which circRNAs enhance or suppress drug resistance in cancers (**Figure 1**), which indicates that circRNAs may function as potential biomarkers and therapeutic targets for cancer.

## Mechanisms of Drug Resistance

### Efflux of Drugs

Drug efflux from cancer cells is a common and important mechanism of resistance or multidrug resistance (MDR), which is inseparable from ATP-binding cassette (ABC) efflux transporters, including MDR-associated protein 2 (MRP2/ABCC2), P-glycoprotein (P-gp/ABCB1), and breast cancer resistance protein (BCRP/ABCG2) (15). ABC efflux transporters, as membrane protein complexes, are often overexpressed in cancer cells and form a defense system against chemotherapeutic drugs as well as a variety of cytotoxic agents, which greatly restricts the effective application of chemotherapy. For example, P-glycoprotein (P-gp) has a wide substrate spectrum that mediates the export of a multitude of drugs, including antibiotics, immunosuppressive agents, and chemotherapeutic drugs (16).

Several researchers have discovered that the function of some circRNAs in drug resistance of cancers is related to ABC efflux transporters. For example, it has been found that circ_0076305 enhances *ABCC1* expression by sponging miR-186-5p, thus regulating cisplatin (CDDP) resistance in non-small cell lung cancer (NSCLC) (17). Similarly, ABCB1 overexpression can reverse the effects of circRNA_103615 silencing on CDDP resistance in NSCLC (18). CircSETD3 upregulates the expression of the ABCG2 transporter by binding to miR-520h, mediating gefitinib to be pumped out of NSCLC cells (19). Both circ_0002060 and circPVT1 contribute to drug resistance in osteosarcoma cells by regulating the expression of *ABCB1* (20, 21). Additionally, the knockdown of circ-CHI3L1.2 downregulates the expression levels of P-gp, *MRP1*, and *GSTP1* and weakens CDDP resistance in osteosarcoma (22). CircPTGR1 and ABCC1 levels are significantly overexpressed in hepatocellular carcinoma (HCC) cells, and circPTGR1 modulates the 5-FU resistance of HCC cells *via* the miR-129-5p/ABCC1 axis (23). Therefore, it is crucial to explore the relationships among circRNAs, ABC efflux transporters, and tolerance to anti-cancer drugs in order to find new therapeutic targets for cancer.

### Apoptosis

The major aim of cancer chemotherapy is to promote the apoptosis of cancer cells and expose them to anti-cancer drugs. The effector phase of apoptosis involves several pro-apoptotic proteins (e.g., BAX, Bak, Bad) and anti-apoptotic proteins (e.g., BCL-2, BCL-XL, BCL-W) (24). However, the genes in cancer cells commonly demonstrate mutations, including apoptotic genes, which often cause dysfunction. This may result in the occurrence of resistance to chemotherapeutics, as any interference that mediates the activation of anti-apoptotic pathways or suppression of pro-apoptotic signal transduction is a potential mechanism of drug resistance (25).

Certain circRNAs have been shown to regulate the apoptosis of drug-resistant cancer cells by modulating pro- or anti-apoptotic proteins. For example, circAMOTL1 can significantly modulate the expression of the Protein Kinase B (AKT) as well as AKT-related pro-apoptotic (BAX and BAK) factors and anti-apoptotic (BCL-2) proteins, thus mediating the paclitaxel (PTX)-resistant effects in breast cancer (26). CircCCDC66 inhibits apoptosis by targeting miR-618 and resists the release of BCL-2, which is an essential regulator of CDDP resistance in gastric cancer (27). Moreover, hsa_circ_0000520 overexpression increases the expression of the BAX protein and reduces the expression of p-PI3K and p-Akt proteins, ultimately reversing the Herceptin resistance of gastric cancer (28). Re-expression of cDOPEY2 decreases the expression of the anti-apoptotic protein Mcl-1 and substantially strengthens the cell lethality of CDDP by augmenting the apoptotic process in CDDP-resistant esophageal squamous cell carcinoma (ESCC) cells (29). The PI3K/Akt/mTOR signaling pathway plays a crucial role in cell cycle regulation, including cell survival, proliferation, and metabolism, and is intimately related to autophagy and apoptosis (30). Based on the existing literature, PI3K-AKT-mTOR signaling is extensively implicated in chemoresistance and drives the process of malignant tumors (31). Silencing of hsa_circ_0000199 deactivates PI3K/Akt/mTOR signaling to promote apoptosis, enhancing triple-negative breast cancer (TNBC) chemosensitivity (32). *PTEN* is an important tumor suppressor gene that encodes a phosphatase protein and resists the PI3K/Akt/mTOR anti-apoptotic pathway as an antagonist (33). It has been reported that *PTEN* expression can be promoted by circPSMC3 by decreasing miR-10a-5p levels, which enhances the esophageal squamous cell carcinoma (ESCC) cells towards gefitinib sensitivity (34).

### TME

TME consists of the stromal cells, immune cells, and extracellular matrix and mounting evidence suggests that the TME plays a crucial role in multiple aspects of tumor progression, particularly in therapeutic resistance (35). Acquired resistance induced by the TME primarily acts as an adaptive response by the host towards pharmacological damage (36). Li et al. explored the combined effects of cancer stem cells (CSCs), circRNA (hsa_circ_0003222), and immune checkpoint blockers in NSCLC malignant behavior as well as drug resistance and found that NSCLC resistance to anti-PD-L1-based therapy could be reduced by silencing hsa_circ_0003222 (37). Additionally, circFGFR1 promotes immune evasion of NSCLC cells and enhances tolerance to anti- PD-1- based therapy by interacting with miR-381-3p and upregulating the expression of C-X-C motif chemokine receptor 4 (CXCR4) (38). Tumor-infiltrating lymphocytes (TILs), an important component of the TME, have attracted increasing attention for therapy resistance in recent years. CircIGF2BP3 expression is negatively associated with the infiltration of CD8^+^ T cells, which induces immune escape from CD8^+^ T cell-mediated killing. Mechanistically, PKP3 upregulated by circIGF2BP3 combines with FXR1 to stabilize OTUB1 mRNA, which increases PD-L1 abundance by promoting its deubiquitination (39). A study on ovarian cancer has suggested that silencing of circ_C20orf11 suppresses extracellular vesicle (EV)-induced macrophage M2 polarization and enhances sensitivity to CDDP *in vivo* (40).

In addition, the interactions between cancer cells and TME also play a pivotal role in epithelial-mesenchymal transition (EMT), which has emerged as a significant cancer cell behavior correlated with metastatic potential and chemoresistance (41). The EMT-associated NF-κB/HER2/STAT3 pathway is crucial in radioresistance of breast cancer stem cells (42). However, the relationship between circRNAs and EMT in drug resistance still needs to be investigated. A recent study has found that the HGF/c-Met pathway regulates the expression of circCCDC66 and SAE2 to promote EMT and chemoresistance in lung adenocarcinoma (LADC) cells (43). In order to overcome this resistance, disruption of EMT-related pathways in tumor cells is desirable.

### Other Mechanisms

Many other drug resistance mechanisms of cancers, including autophagy, glycolysis, dysfunction of DNA damage repair, altered drug targets, decreased drug influx, and so forth are known (14). However, the relationship between circRNAs and the above mentioned drug resistance mechanisms are still being investigated. Autophagy plays a crucial role in tumorigenesis, progression, and therapeutic intervention in cancers (44). A previous study has found that the knockdown of autophagy-related genes (*ATG5* and *ATG7*) enhances therapeutic cell killing, indicating that autophagy may promote the acquired resistance of cancer cells to chemotherapeutics (45). Hsa_circ_0085131 and hsa_circ_0092276 serve as competitive endogenous RNAs (ceRNAs) to elevate autophagy-associated factor *ATG7* expression, thus enhancing CDDP resistance in NSCLC and doxorubicin (DOX) resistance in breast cancer (46, 47). Tumor cells often switch to glycolysis, even under aerobic conditions, and components of the glycolytic pathway, including transporters, enzymes, and metabolites, are involved in inducing drug resistance (48). Some circRNAs mediate chemotherapy resistance by modulating glycolysis in tumor cells. For instance, circ_0008928 silencing enhances CDDP sensitivity in CDDP-resistant NSCLC cells and impedes NSCLC progression and glycolysis by upregulating miR-488 expression and downregulating HK2 expression (49). Similarly, the CDDP-resistance mechanism of circAKT3 and circRNA-FOXO3 is involved in disturbing the glycolysis balance (50, 51). CDDP sensitivity is suppressed partly by circAKT3 by modulating the miR-516b-5p/STAT3 axis in lung cancer cells, whereas CDDP sensitivity is promoted by circRNA-FOXO3 *via* the miR-543/Foxo3 axis in NSCLC cells. The hypoxia-induced exosomal circZNF91 transferred to normoxic pancreatic cancer cells can interact with miR-23b-3p and upregulate the expression of the deacetylase sirtuin1 (*SIRT1*), thereby enhancing the deacetylation-dependent stability of HIF-1α, resulting in gemcitabine (GEM) resistance and glycolysis-induced chemoresistance (52). DNA damage is a relatively common and vital cellular event that is implicated in mutations, metabolic dysfunction, cellular or organismic death, and tumorigenesis (53). Upregulation of processes such as DNA damage tolerance (DDT) and DNA damage response (DDR) is advantageous to cancer cells because it allows them to resist damage lesions (54). A large number of processes in cancers activate cellular DDR to remove or repair DNA lesions (54). Dysregulation of DDR is often the route by which tumor cells evade chemotherapy (55). A recent study found that circAKT3 had an impact on DDR in gastric cancer cells and might promote CDDP resistance in gastric cancer through the DDR and PI3K/AKT pathways (56). Alterations in chemotherapeutic targets can have a great influence on drug resistance. Alterations in DNA topoisomerase-II (Topo-II) activity, such as topo-II mutations or downregulation of topo-II protein, can result in resistance to topo-II-targeted drugs, including anthracyclines (57). As for drug influx, the mechanism by which only a small amount of chemotherapeutics enters cells has been elucidated. For instance, methotrexate (MTX), a dihydrofolate reductase (DHFR) inhibitor, enters the cell predominantly *via* the decreased folate carrier (RFC) (58). Decreased expression and inactivating alterations of the RFC are documented mechanisms of MTX resistance (59). Taken together, diverse mechanisms of drug resistance have been discovered, however more detailed mechanisms of chemoresistance are still largely unknown and require further investigation.

## CircRNAs and Drug Resistance

### CircRNAs and Lung Cancer Drug Resistance

According to the global cancer statistics of 2020, lung cancer remains a major cause of cancer-related deaths (60). CDDP is one of the most effective anti-cancer drugs and is extensively used in the clinic; however, the development of CDDP resistance seriously hinders the therapeutic effect of cancer (61). The involvement of circRNAs in drug resistance in lung cancer is shown in **Table 1**. Accumulating evidence has demonstrated that circRNAs are implicated in CDDP resistance in lung cancer. Knockdown or silencing of certain circRNAs suppresses CDDP resistance in NSCLC such as circ-RNF121, circ_0076305, circRNA_103615, circ_0008928, hsa_circRNA_103809, circ-PRMT5, and circ_0007385 (17, 18, 49, 62–65). Additionally, circAKT3 inhibits glycolysis balance and enhances CDDP resistance, whereas circRNA-FOXO3 accelerates glycolysis and promotes CDDP sensitivity (50, 51). Both hsa_circ_0014235 and hsa_circ_0096157 overexpression intensifies the CDDP resistance and facilitates the malignancy of NSCLC cells, including proliferation, migration, and invasion (66, 67). circRNA_100565 is correlated with a poor prognosis of NSCLC, and circRNA_100565 deletion mitigates CDDP resistance, which can be attenuated by miR-377-3p inhibition or ADAM28 overexpression (68). circ_PIP5K1A regulates CDDP sensitivity *via* the miR-493-5p/ROCK1 axis, and circ_0085131 enhances NSCLC cell drug resistance by targeting miR-654-5p to upregulate *ATG7* expression (46, 69). Additionally, the sensitivity of CDDP can be promoted by the knockdown of circ-ABCB10 in lung cancer cells (70). However, circ_0000079 overexpression disturbs the formation of the FXR1/PRCKI complex by regulating FXR1, thereby suppressing cell invasion and CDDP resistance in NSCLC (71).

**Table 1:** Lung cancer drug resistance related circRNAs.

CircRNA	Source	Expression	Sponging miRNAs	Targets and Pathways	Resistant Drugs	Cancer Type	Reference
circ_0076305	exosomes	up	miR-186-5p	ABCC1	cisplatin	NSCLC	(17)
circRNA_103615	N/A	up*	N/A	ABCC1	cisplatin	NSCLC	(18)
circ_0008928	exosomes	up	miR-488	HK2	cisplatin	NSCLC	(49)
circ-RNF121	N/A	up	miR-646	SOX4	cisplatin	NSCLC	(62)
hsa_circRNA_103809	N/A	up	miR-377-3p	GOT1	cisplatin	NSCLC	(63)
circ-PRMT5	N/A	up	miR-4458	REV3L	cisplatin	NSCLC	(64)
circ_0007385	N/A	up	miR-519d-3p	HMGB1	cisplatin	NSCLC	(65)
circAKT3	N/A	up*	miR-516b-5p	STAT3	cisplatin	lung cancer	(50)
circRNA-FOXO3	N/A	up*	microRNA-543	Foxo3	cisplatin	NSCLC	(51)
hsa_circ_0014235	exosomes	up*	miR-520a-5p	CDK4	cisplatin	NSCLC	(66)
hsa_circ_0096157	N/A	up	N/A	N/A	cisplatin	NSCLC	(67)
circRNA_100565	N/A	up	miR-377-3p	ADAM28	cisplatin	NSCLC	(68)
circ_PIP5K1A	N/A	up	miR-493-5p	ROCK1	cisplatin	NSCLC	(69)
hsa_circ_0085131	N/A	up	miR-654-5p	ATG7	cisplatin	NSCLC	(46)
circ-ABCB10	N/A	up*	miR-556-3p	AK4	cisplatin	lung cancer	(70)
circ_0000079	N/A	down	N/A	FXR1	cisplatin	NSCLC	(71)
circ_0001821	N/A	up*	miR-526b-5p	GRK5	paclitaxel	NSCLC	(72)
circ_ZFR	N/A	up	miR-195-5p	KPNA4	paclitaxel	NSCLC	(73)
circ_0011292	N/A	up	miR-379-5p	TRIM65	paclitaxel	NSCLC	(74)
hsa_circ_0002874	N/A	up	miR1273f	MDM2/P53 pathway	paclitaxel	NSCLC	(75)
hsa_circ_0030998	N/A	down	miR-558	N/A	paclitaxel	lung cancer	(76)
circ_0002483	N/A	down*	miR-182-5p	GRB2, FOXO1, and FOXO3	Taxol	NSCLC	(77)
circ_0014130	N/A	up	miR-545-3p	YAP1	docetaxel	NSCLC	(78)
circ_0003998	N/A	up	miR-136-5p	CORO1C	docetaxel	NSCLC	(79)
hsa_circ_0005909	cytoplasm	up*	miRNA-338-3p	SOX4	adriamycin	NSCLC	(80)
circASK1	N/A	down	ASK1	N/A, ASK1/JNK/p38 signaling	gefitinib	LUAD	(81)
circSETD3	N/A	up	miR-520h	ABCG2	gefitinib	NSCLC	(18)
circRNA_102481	exosomes	up	miR-30a-5p	ROR1	EGFR-TKIs	NSCLC	(82)
hsa_circ_0003222	N/A	up*	miR-527	N/A	anti-PD-L1	NSCLC	(37)
circFGFR1	N/A	up*	miR-381-3p	CXCR4	anti-PD-1	NSCLC	(38)
circIGF2BP3	N/A	up*	miR-328-3p, miR-3173-5p	PKP3	anti-PD-1	NSCLC	(39)

N/A, Not Applicable.

*The expression of circRNA upregulated only in cancer cells, others (without *) upregulated in cancer drug-resistant cells or both.

PTX is also an anti-neoplastic agent widely used to treat several solid tumor types, including lung cancer. Knockdown of circ_0001821 and circ_ZFR reduces the proliferation and metastasis ability but enhances PTX sensibility and apoptosis of NSCLC cells by downregulating the expression of GRK5 and KPNA4 by sponging related miRNAs (72, 73). Similarly, circ_0011292 modulates the miR-379-5p/TRIM65 axis to promote tumorigenesis and enhance PTX resistance in NSCLC (74). It has also been found that hsa_circ_0002874 downregulation can reverse the PTX resistance of NSCLC and induce apoptosis by regulating the miR1273f/MDM2/P53 signaling pathway (75). In contrast to the above circRNAs, circ_0030998 and circ_0002483 overexpression decreases the malignant behavior of lung cancer cells and enhances their sensitivity to PTX by targeting miR-558 and miR-182-5p, respectively (76, 77).

Du et al. explored a special circ_0014130-miR-545-3p-YAP1 pathway in the modulation of drug resistance and malignant behaviors of docetaxel (DTX)-resistant NSCLC cells (78). circ_0003998 knockdown suppresses colony formation and facilitates apoptosis and DTX sensitivity by sponging miR-136-5p to control CORO1C expression in DTX-resistant NSCLC cells (79). It has also been found that circ_0005909 knockdown resists the proliferation, metastasis, and drug resistance of NSCLC cells (80). Moreover, a novel protein encoded by circASK1 activates ASK1-dependent apoptosis, thereby ameliorating gefitinib resistance in LUAD (81). It has been shown that circSETD3 overexpression interferes with gefitinib sensitivity, and circSETD3 interacts with miR-520h and ABCG2 to reduce the intracellular concentration of gefitinib (19).

Exosomes are small extracellular vesicles that play essential roles in immunity, signal transduction, tumor treatment, and drug resistance (83). Yang et al. suggested that tumor-derived exosomal circRNA_102481 participated in EGFR-TKI resistance by sponging microRNA-30a-5p to modulate ROR1 in NSCLC (82). As for immune checkpoint inhibitors, hsa_circ_0003222 and circFGFR1 can promote NSCLC resistance to anti-PD-L1-based and anti-PD-1-based therapies, respectively (37, 38). Furthermore, suppression of circIGF2BP3/PKP3 promotes the therapeutic efficacy of anti-PD-1 in a Lewis LADC mouse model (39).

### CircRNAs and Breast Cancer Drug Resistance

It was reported that there were 2.3 million new cases of breast cancer in female patients, which became the most generally diagnosed cancer, exceeding lung cancer in 2020 (60). Multiple circRNAs are shown to be correlated with breast cancer resistance (**Table 2**). The resistance to adriamycin (ADM), namely DOX, is closely correlated with therapeutic efficacy in patients with breast cancer and their prognosis (93). The knockdown of circ_0085495, circ_0001667, and circ_0006528 attenuates ADM resistance by related sponging miRNAs, becoming promising therapeutic targets for overcoming ADM resistance in patients with breast cancer (84–86). Similarly, hsa_circ_0092276, which sponges miR-384, regulates *ATG7* expression and promotes DOX resistance in breast cancer (47). Additionally, circUBE2D2 depletion induces a tumor-suppressive effect and suppression in DOX resistance, which has been greatly impaired upon miR512-3p downregulation or CDCA3 overexpression (87).

**Table 2:** Breast cancer drug resistance related circRNAs.

CircRNA	Source	Expression	Sponging miRNAs	Targets and Pathways	Resistant Drugs	Cancer Type	Reference
circ_0085495	cytoplasm	up	miR-873-5p	integrin β1	adriamycin	breast cancer	(84)
circ_0001667	N/A	up	miR-4458	NCOA3	adriamycin	breast cancer	(85)
circ_0006528	N/A	up	miR-1236-3p	CHD4	adriamycin	breast cancer	(86)
hsa_circ_0092276	N/A	up	miR-348	ATG7	doxorubicin	breast cancer	(47)
circUBE2D2	N/A	up*	miR-512-3p	CDCA3	doxorubicin	TNBC	(87)
circ-MMP11	exosomes	up	miR-153-3p	ANLN	lapatinib	breast cancer	(88)
circFAT1	N/A	up	miR-525-5p	SKA1, Notch and Wnt pathway	oxaliplatin	breast cancer	(89)
hsa_circ_0000199	unclear	up	miR-613 and miR-206	PI3K/Akt/mTOR signaling	cisplatin, adriamycin, paclitaxel, gemcitabine	TNBC	(32)
circ-RNF111	N/A	up	miR-140-5p	E2F3	paclitaxel	breast cancer	(90)
circAMOTL1	N/A	up	N/A	AKT pathway	paclitaxel	breast cancer	(26)
circFBXL5	N/A	up	miR-216b	HMGA2	5-fluorouracil	breast cancer	(91)
hsa_circ_0025202	N/A	down	miR-197-3p	HIPK3	tamoxifen	breast cancer	(92)

N/A, Not Applicable.

*The expression of circRNAs only upregulated in cancer cells, others (without *) upregulated in cancer drug-resistant cells or both.

Lapatinib resistance is promoted by circ-MMP11 in breast cancer cells, and mechanically circ-MMP11 regulates ANLN expression by sponging miR-153-3p (88). CircFAT1 facilitates oxaliplatin (OX) resistance in breast cancer by regulating miR-525-5p/SKA1, and the Notch and Wnt pathways can be activated by SKA1, which has been identified by rescue assays, GSEA, and western blotting (89). Li et al. found that silencing hsa_circ_0000199 contributed to TNBC chemosensitivity to multiple drugs (32). In their study, the TNBC cell lines in the si-hsa_circ_0000199 group are prone to become sensitive to chemotherapeutic drugs, including CDDP, adriamycin, paclitaxel, and gemcitabine (GEM). It has also been found that circ-RNF111 increases PTX resistance in breast cancer by elevating E2F3 *via* sponging miR-140-5p (90). CircAMOTL1 overexpression reduces apoptosis and enhances the invasion of breast cancer cells exposed to PAX (26). Furthermore, 5-FU resistance is promoted by circFBXL5 in breast cancer *via* the miR-216b/HMGA2 axis (91). Additionally, hsa_circ_0025202 overexpression impedes tumor growth and promotes tamoxifen sensitivity, while miR-197-3p overexpression facilitates cell malignancy and TAM resistance in breast cancer (92).

### CircRNA and Gastric Cancer Drug Resistance

Gastric cancer (GC) is the fifth most common malignant tumor and is the fourth leading cause of cancer-related deaths (60, 94). The 5-year survival rate of advanced GC is approximately 20% (95). Platinum is a basic first-line chemotherapy drug for advanced GC (95). Studies on the mechanisms of circRNAs and resistance to CDDP are presented in **Table 3**. Exosomal circ-PVT1 regulates invasion, autophagy, and apoptosis and promotes CDDP resistance *via* the miR-30a-5p/YAP1 axis in GC cells, indicating that exosomal circ-PVT1 may be a valuable therapeutic target in GC (96). Additionally, the sensitivity of GC to CDDP is increased by knockdown of circ_ASAP2 or hsa_circ_0081143, which also represses the progression of GC by acting as ceRNAs (97, 98). It has been found that circAKT3 contributes to CDDP resistance in GC by enhancing DNA damage repair and hindering GC cell apoptosis (56). CircCCDC66 and circDONSON have been shown to induce CDDP resistance in GC by targeting the related miRNA and gene (27, 99). All the above studies demonstrate that certain circRNAs are overexpressed in CDDP-resistant GC and may serve as biomarkers for poor prognosis.

**Table 3:** Gastric cancer drug resistance related circRNAs.

CircRNA	Source	Expression	Sponging miRNAs	Targets	Resistant Drugs	Cancer Type	Reference
circ-PVT1	exosomes	up	miR-30a-5p	YAP1	cisplatin	GC	(96)
circ_ASAP2	N/A	up	miR-330-3p	NT5E	cisplatin	GC	(97)
hsa_circ_0081143	N/A	up*	miR-646	CDK6	cisplatin	GC	(98)
circAKT3	N/A	up	miR-198	PIK3R1	cisplatin	GC	(56)
circCCDC66	N/A	up	miR-618	BCL2	cisplatin	GC	(27)
circDONSON	N/A	up	miR-802	BMI1	cisplatin	GC	(99)
circ_0001017	N/A	down	miR-543	PHLPP2	cisplatin	GC	(100)
circCUL2	cytoplasm	down	miR-142-3p	ROCK2	cisplatin	GC	(101)
hsa_circ_0032821	exosomes	up	miR-515-5p	SOX9	oxaliplatin	GC	(102)
hsa_circ_0000520	N/A	down	N/A	N/A	herceptin	GC	(28)

N/A, Not Applicable.

*The expression of circRNAs only upregulated in cancer cells, others (without *) upregulated in cancer drug-resistant cells or both.

In contrast, Zhang et al. illustrated that upregulated expression of circ_0001017 inhibited malignant biological behaviors of GC and promoted CDDP sensitivity of CDDP-resistant GC cells partially *via* the miR-543/PHLPP2 axis (100). Similarly, circCUL2, acting as a tumor suppressor, enhances CDDP sensitivity by miR-142-3p/ROCK2-mediated autophagy (101). Exosomal circ_0032821 facilitates OXA resistance in OXA-sensitive GC cells by sponging miR-515-5p to enhance SOX9 expression (102). In addition, Herceptin resistance of GC cells can be reversed by hsa_circ_0000520 overexpression through inhibition of the PI3K-Akt signaling pathway (28).

### CircRNAs and Osteosarcoma Drug Resistance

Osteosarcoma is the most widespread primary bone tumor affecting children and adolescents, and effective chemotherapeutic regimens include the combination of high-dose MTX, DOX, and CDDP (103). As shown in **Table 4**, multiple circRNAs are associated with osteosarcoma drug resistance. Circ_0081001 knockdown promotes MTX sensitivity of osteosarcoma cells through the suppression of miR-494-3p-mediated upregulation of TGM2 (104). Similarly, hsa_circ_0000073 promotes malignant behaviors, including proliferation, invasion, and migration, and facilitates MTX resistance of osteosarcoma cells by sponging miR-145-5p and miR-151-3p to upregulate NRAS (105).

**Table 4:** Osteosarcoma drug resistance related circRNAs.

CircRNA	Source	Expression	Sponging miRNAs	Targets	Resistant Drugs	Cancer Type	Reference
circ_0081001	N/A	up	miR-494-3p	TGM2	Methotrexate	osteosarcoma	(104)
hsa_circ_0000073	N/A	up	miR-145-5p and miR-151-3p	NRAS	Methotrexate	osteosarcoma	(105)
hsa_circ_0004674	N/A	up	miR-342-3p	FBN1	doxorubicin	osteosarcoma	(106)
circ_0001721	N/A	up	miR-758	TCF4	doxorubicin	osteosarcoma	(107)
circSAMD4A	N/A	up	miR-218-5p	KLF8	doxorubicin	osteosarcoma	(108)
circ_0002060	N/A	up	miR-198	ABCB1	doxorubicin	osteosarcoma	(20)
circ_0003496	N/A	up	miR-370	KLF12	doxorubicin	osteosarcoma	(109)
circPVT1	N/A	up	N/A	ABCB1	doxorubicin and cisplatin	osteosarcoma	(20)
circ-CHI3L1.2	N/A	up	miR-340-5p	LPAATβ	cisplatin	osteosarcoma	(22)
hsa_circ_103801	exosomes	up	N/A	N/A	cisplatin	osteosarcoma	(110)
circTADA2A	N/A	up	miR-129-5p	TRPS1, YAPS	cisplatin	osteosarcoma	(111)

N/A, Not Applicable.

DOX resistance of osteosarcoma is facilitated by hsa_circ_0004674 through the Wnt/β-catenin pathway *via* modulation of the miR-342-3p/FBN1 axis (106). Moreover, some circRNAs potentiate DOX resistance and facilitate the progression of osteosarcomas, such as circ_0001721, circSAMD4A, circ_0002060, and circ_0003496 (20, 107–109). Zhu et al. report that resistance to DOX and CDDP can be weakened by suppressing circPVT1 expression in osteosarcoma cells (21). It has been reported that circ-CHI3L1.2 knockdown promotes apoptosis and attenuates resistance of CDDP-resistant osteosarcoma cells (22). Additionally, exosomal hsa_circ_103801 can intensify the facilitating effect of exosomes on the chemoresistance of osteosarcoma cells to CDDP (110). Similarly, circTADA2A can target miR-129-5p, which is competitively bound to TRPS1 and YAP1, thereby regulating osteosarcoma cell proliferation and CDDP resistance (111).

### CircRNA and Glioma Drug Resistance

Glioma is the most common form of aggressive intracranial tumors and is characterized by a high rate of mortality, metastasis, and drug resistance (112). Here, we summarized the contribution of circRNAs to chemoresistance in glioma patients (**Table 5**). It has been shown that exosomal hsa_circ_0042003, mediated by heparanase transfers from temozolomide (TMZ)-resistant glioma cells to drug-sensitive cells, which contributes to TMZ resistance in glioma (113). In glioma, exosomal circ_0072083 modulates NANOG and ALKBH5 by targeting miR-1252-5p and demethylation to control TMZ resistance (114). Hsa_circ_0110757 and circ_0005198 facilitate TMZ resistance and inhibit glioma cell apoptosis through the miR-1298-5p/ITGA1 and miR-198/TRIM14 axis, respectively (115, 116). Consistent with these findings, downregulation of circ-VPS18, hsa_circ_0000936, circHIPK3, and circ CEP128 improve TMZ sensitivity and repress glioma progression (117–120). The highlight is that circHIPK3 downregulation inhibits the PI3K/AKT signaling pathway partly through the miR-524-5p/KIF2A axis (119). Similarly, in TMZ-resistant glioma, exosomal circ-HIPK3 can regulate the miR-421/ZIC5 axis to promote cell progression and TMZ resistance (123). circASAP1 overexpression promotes glioblastoma cell proliferation and TMZ resistance *via* the circASAP1/miR-502-5p/NRAS regulatory network, indicating that circASAP1 is a potential target for TMZ-resistant glioblastoma therapy (121). circ_0008344 downregulation impedes glioma growth and functions on the miR-433-3p/RNF2 axis to promote radiosensitivity in glioma (122).

**Table 5:** Glioma drug resistance related circRNAs.

CircRNA	Source	Expression	Sponging miRNAs	Targets	Resistant Drugs	Cancer Type	Reference
hsa_circ_0042003	exosomes	up	N/A	N/A	Temozolomide	glioma	(113)
circ_0072083	exosomes	up	miR-1252-5p	NANOG	Temozolomide	glioma	(114)
Hsa_circ_0110757	N/A	up	miR-1298-5p	ITGA1	Temozolomide	glioma	(115)
circ_0005198	N/A	up	miR-198	TRIM14	Temozolomide	glioma	(116)
circ-VPS18	N/A	up	miR-370	RUNX1	Temozolomide	glioma	(117)
hsa_circ_0000936	N/A	up	miR-1294	N/A	Temozolomide	glioma	(118)
circHIPK3	N/A	up	miR-524-5p	KIF2A	Temozolomide	glioma	(119)
circ CEP128	N/A	up	miR-145-5p	N/A	Temozolomide	glioma	(120)
circ-HIPK3	exosomes	up	miR-421	ZIC5	Temozolomide	glioma	(119)
circASAP1	N/A	up	miR-502-5p	NRAS	Temozolomide	glioblastoma	(121)
circ_0008344	N/A	up	miR-433-3p	RNF2	radiotherapy	glioma	(122)

N/A, Not Applicable.

### CircRNAs and Ovarian Cancer Drug Resistance

Ovarian cancer is one of the most common gynecologic malignant tumors, and conventional treatment is mainly limited to chemoresistance (124). **Table 6** displays the chemoresistance-related circRNAs in ovarian cancer. Both circ_C20orf11 and circulating exosomal circFoxp1 can confer CDDP resistance in ovarian cancer cells (40, 125). In contrast, Cdr1as improves sensitivity to CDDP in ovarian cancer by modulating the miR-1270/SCAI axis (126).

**Table 6:** Ovarian cancer drug resistance related circRNAs.

CircRNA	Source	Expression	Sponging miRNAs	Targets	Resistant Drugs	Cancer Type	Reference
circ_C20orf11	N/A	up*	miR-527	YWHAZ	cisplatin	ovarian cancer	(40)
circFoxp1	exosomes	up	miR-22 and miR-150-3p	CEBPG and FMNL3	cisplatin	ovarian cancer	(125)
circRNA Cdr1as	N/A	down	miR-1270	SCAI	cisplatin	ovarian cancer	(126)
circRNA_0006404	N/A	down	miR-346	DKK3	Docetaxel	ovarian cancer	(127)
circRNA_0000735	N/A	up	miR-526b	DKK4	Docetaxel	ovarian cancer	(127)
circ_CELSR1	N/A	up	miR-149-5p	SIK2	paclitaxel	ovarian cancer	(128)
circCELSR1	N/A	up	miR-1252	FOXR2	paclitaxel	ovarian cancer	(129)
circTNPO3	N/A	up	miR-1299	NEK2	paclitaxel	ovarian cancer	(130)

N/A, Not Applicable.

*The expression of circRNAs only upregulated in cancer cells, others (without *) upregulated in cancer drug-resistant cells or both.

The downregulation of circRNA_0000735 and upregulation of circRNA_0006404 can suppress the expression of p-GP, causing DTX treatment tolerance (127). Two studies demonstrated that circ_CELSR1 was upregulated in PTX-resistant ovarian cancer cells, and circ_CELSR1 silencing impeded PTX resistance in ovarian cancer *in vivo* (128, 129). Meanwhile, one study revealed that the inhibition of circCELSR1 also resulted in ovarian cancer cell G_0_/G_1_ arrest and the promotion of apoptosis (129). Similarly, circTNPO3 upregulates NEK2 expression by sponging miR-1299 to enhance PTX resistance in ovarian cancer cells (130).

### CircRNA and Drug Resistance of Other Cancers

We also summarized a number of studies on circRNAs and drug resistance in other cancers, including hepatocellular carcinoma (HCC), colorectal cancer (CRC), esophageal cancer (EC), pancreatic cancer (PC), and some urinary system tumors (**Table 7**). As we all know, tolerance to chemotherapeutics is the pivotal cause of recurrence and poor prognosis in colorectal cancer patients. It has been shown that circ_0000338 knockdown sensitizes 5-FU-resistant CRC cells to 5-FU by promoting apoptosis and hindering proliferation (131). Exosomal circ-FBXW7 leads resistant cells sensitive to OX-induced apoptosis, inhibits OX-induced epithelial-mesenchymal transition, and suppresses OX efflux (132). In contrast, hsa_circ_0005963 is transferred by exosomes from OX-resistant CRC cells to OX-sensitive cells, resulting in glycolysis and drug resistance by enhancing PKM2 expression (133). A recent study suggested that circ_0020095 acts as a miR-487a-3p sponge to promote CDDP resistance by increasing the expression of SOX9 (134). In DOX-resistant CRC cells, circCSPP1 knockdown improves DOX sensitivity, attenuates cell malignant behaviors, and induces apoptosis through the miR-944/FZD7 axis (135).

**Table 7:** Drug resistance related circRNAs of other cancers.

CircRNA	Source	Expression	Sponging miRNAs	Targets and Pathways	Resistant Drugs	Cancer Type	Reference
circ_0000338	exosome	up	miR-217 and miR-485-3p	N/A	5-fluorouracil	CRC	(131)
circ-FBXW7	exosome	down	miR-18b-5p	N/A	oxaliplatin	CRC	(132)
hsa_circ_0005963	exosome	up	miR-122	PKM2	oxaliplatin	CRC	(133)
circ_0020095	N/A	up*	miR-487a-3p	SOX9	cisplatin	colon cancer	(134)
circCSPP1	N/A	up	miR-944	FZD7	doxorubicin	CRC	(135)
circPTGR1	N/A	up*	miR-129-5p	ABCC1	5-fluorouracil	HCC	(23)
circARNT2	N/A	up	miR-155-5p	PDK1	cisplatin	HCC	(136)
circRNA_102272	N/A	up*	miR-326	RUNX2	cisplatin	HCC	(137)
circRNA-SORE	exosomes	up	N/A	YBX1	sorafenib	HCC	(138)
circRNA-SORE	N/A	up	miR-103a-2-5p and miR-660-3p,	Wnt/β-cateninpathway	sorafenib	HCC	(139)
two novel circRNAs^△^	N/A	up	miR-19a-3p and miR-145-5p	N/A	Gemcitabine	pancreatic cancer	(140)
circZNF91	exosomes	up*	miR-23b-3p	SIRT1	Gemcitabine	pancreatic cancer	(52)
circ_0000337	exosomes	up	miR-377-3p	JAK2	cisplatin	esophageal cancer	(141)
circRNA-DOPEY2	N/A	down	N/A	N/A	cisplatin	ESCC	(29)
circ_0006168	N/A	up	miR-194-5p	JMJD1C	Taxol	esophageal cancer	(142)
circPSMC3	N/A	down	miR-10a-5p	PTEN	gefitinib	ESCC	(34)
circFNTA	N/A	up*	miR-370-3p	FNTA, KRAS signaling	cisplatin	bladder cancer	(143)
circELP3	cytoplasm	up*	N/A	N/A	cisplatin	bladder cancer	(144)
circ0008399	N/A	up*	WTAP	TNFAIP3	cisplatin	bladder cancer	(145)
hsa_circ_0000285	N/A	down	N/A	N/A	cisplatin	bladder cancer	(146)
hsa_circ_0035483	N/A	up*	miR-335	CCNB1	gemcitabine	RCC	(147)

N/A, Not Applicable.

^△^chr14:101402109-101464448+ and chr4:52729603-52780244+.

*The expression of circRNAs upregulated only in cancer cells, others (without *) upregulated in cancer drug-resistant cells or both.

As for HCC, the knockdown of circPTGR1 and circARNT2 induces apoptosis of HCC cells and inhibits 5-FU and CDDP resistance, respectively (23, 136). Moreover, circRNA, as a ceRNA, suppresses the activity of miR-326 to upregulate RUNX2 expression and enhance CDDP resistance (137). Two studies elaborate the mechanism of circRNA-SORE in sorafenib resistance in HCC from different perspectives. One study reveals that circRNA-SORE inhibits PRP19-mediated YBX1 degradation, thus affecting the expression of downstream gene targets of YBX1, including ERK, AKT, c-Myc, Raf1, and TGF-β1 (138). Another study demonstrates that circRNA-SORE interacts with miR-103a-2-5p and miR-660-3p by serving as a microRNA sponge, thus activating the Wnt/β-catenin pathway and leading to sorafenib resistance (139). In addition, Huang et al. confirm that overexpression of circMET can promote HCC development and immune tolerance through the miR-30-5p/Snail/DPP4/CXCL10 axis (148).

An increasing number of studies have elucidated the molecular mechanism of circRNAs in the initiation and progression of PC; however, studies on drug resistance mechanisms are in their preliminary exploration stage (149). For example, PC drug-resistant cells (PANC-1-GR) increase sensitivity to GEM after silencing two novel circRNAs (chr14:101402109-101464448+ or chr4:52729603-52780244+) (140). Exosomal circZNF91 transferred to normoxic PC cells can result in GEM and glycolysis chemoresistance (52).

As early as 2016, researchers utilized circRNA microarray to discover that some circRNAs are distinguished in the radioresistant esophageal cell line and the parental cell line, indicating that these dysregulated circRNAs are involved in radiation resistance (150). In esophageal cancer cells, CDDP resistance can be increased by exosomal circ_0000337, which regulates the miR-377-3p/JAK2 axis (141). cDOPEY2 promotes the ubiquitination and degradation of CPEB4 to inhibit CPEB4-mediated Mcl-1 translation, thereby alleviating CDDP resistance (29). Moreover, knockdown of circ_0006168 or JMJD1C plays a crucial role in inhibiting cell proliferation, invasion, and migration and promoting apoptosis, which accelerates the taxol sensitivity of Esophageal squamous cell carcinoma (ESCC) *in vitro* (142). In contrast, circPSMC3 overexpression inhibits miR-10a-5p expression and increases the sensitivity of ESCC cells to gefitinib (34).

Research on circRNAs in the drug resistance of urinary system tumors is also in its initial stages. It is an AR-mediated ADAR2/circFNTA/miR-370-3p/FNTA pathway that stimulates KRAS to change bladder cancer cell invasion and chemosensitivity to CDDP (143). Hypoxia-elevated circELP3 facilitates CDDP resistance in bladder cancer cells (144). circ0008399 promotes the formation of the WTAP/METTL3/METTL14 m^6^A methyltransferase complex and increases the expression of TNFAIP3 in an m^6^A-dependent manner. Additionally, the high expression of circ0008399 and WTAP is correlated with poor prognosis in patients with bladder cancer (145). A study reveals that hsa_circ_0000285 is expressed at significantly lower levels in both bladder cancer tissues and CDDP-resistant patients, but the detailed mechanism of hsa_circ_0000285 in chemosensitivity needs further exploration (146). In addition, hsa_circ_0035483 contributes to GEM-induced autophagy and promotes resistance of renal clear cell carcinoma (RCC) to GEM by regulating the hsa-miR-335/CCNB1 axis (147).

## Conclusions and Future Perspectives

Currently, resistance to chemotherapeutic drugs has become an urgent problem impeding the treatment of various cancers. As a novel RNA class, an increasing number of circRNAs have been confirmed to be related to the chemoresistance of cancers. Most circRNAs function as miRNA sponges and form the circRNAs/miRNAs/mRNAs regulatory axis to regulate drug resistance or sensitivity of cancers. The sensitivity of cancer cells to chemotherapeutic drugs can be enhanced by silencing circRNAs that are upregulated in drug-resistant cancer cells or by overexpressing circRNAs that are downregulated in drug-resistant cancer cells.

The underlying mechanisms of chemoresistance-related circRNAs include the efflux of drugs, apoptosis, glycolysis, intervention with the TME, autophagy, and dysfunction of DNA damage repair, among others, which still requires further exploration. In addition, multiple pathways are involved in circRNA-modulated drug resistance in cancers including P53 signaling, KRAS signaling, the Wnt/β-catenin pathway, and the PI3K/Akt/mTOR pathway. New challenges owing to different chemotherapies are inevitable, demanding further elucidation of the involved signaling networks.

Taken together, it is still a challenge to select the key target for the treatment of malignancies from a large number of candidate circRNAs. Most studies on circRNAs and drug resistance are limited to *in vitro* and *in vivo* experiments. Therefore, long-term follow-up of patients and analysis of the relationship between circRNAs and drug resistance are warranted in this regard. The drug resistance of cancers may be associated with a systematic and comprehensive regulatory network comprising circRNAs, miRNAs, and target mRNAs. More research is required to elucidate the concrete molecular mechanisms between circRNAs and drug resistance in cancers and to survey the role of circRNAs in clinical practice in the future.

## Author Contributions

Xin-YL, QZ, and JG reviewed literature and originally drafted the manuscript. PZ, HL, Z-BT, and C-PZ contributed to editing and embellished the manuscript. Xia-YL approved the final version of the manuscript. All authors contributed to the article and approved the submitted version.

## Funding

The study was supported by the National Natural Science Foundation (No. 81802777), the “Clinical medicine + X” scientific research project of Affiliated Hospital of Qingdao University, and Qingdao Chinese Medicine Technology Project (2021-zyym26).

## Conflict of Interest

The authors declare that the research was conducted in the absence of any commercial or financial relationships that could be construed as a potential conflict of interest.

## Publisher’s Note

All claims expressed in this article are solely those of the authors and do not necessarily represent those of their affiliated organizations, or those of the publisher, the editors and the reviewers. Any product that may be evaluated in this article, or claim that may be made by its manufacturer, is not guaranteed or endorsed by the publisher.
